# A fully human streptolysin O-neutralizing monoclonal antibody improves survival in a murine model of group A streptococcus necrotizing soft tissue infection

**DOI:** 10.1128/aac.00575-26

**Published:** 2026-07-17

**Authors:** Emily Price, Anyauba Nmaju, Sabrina Faozia, Cheri L. Lamb, Eric R. McIndoo, Sydney Catlin, Marcelo Ayllon, Dylan J. Breuer, Bryan Sanders, Daniel Fologea, Dennis L. Stevens, Amy E. Bryant, Sarah E. Hobdey

**Affiliations:** 1 Boise VA Medical Center 20005, Boise, Idaho, USA; 2 Idaho State University 212907, Meridian, Idaho, USA; 3 Idaho Veterans Research and Education Foundation, Boise, Idaho, USA; 4 Idaho State University 6640, Pocatello, Idaho, USA; 5 University of Washington School of Medicine 12353, Seattle, Washington, USA; 6 Baylor University 14643, Waco, Texas, USA; 7 Brigham Young University-Idaho 6860, Rexburg, Idaho, USA; 8 Boise State University 1791, Boise, Idaho, USA; 9 Idaho College of Osteopathic Medicine 518097, Meridian, Idaho, USA; Columbia University Irving Medical Center, New York, New York, USA

**Keywords:** *Streptococcus pyogenes*, necrotizing soft tissue infections, streptolysin O, monoclonal antibodies, toxin neutralization

## Abstract

Necrotizing soft tissue infections (NSTIs) caused by *Streptococcus pyogenes* (group A streptococcus [GAS]) are rapidly progressive, toxin-driven infections for which no targeted antitoxin therapies exist. Streptolysin O (SLO), a cholesterol-dependent cytolysin, is a key mediator of tissue injury, vascular occlusion, and immune dysfunction and, thus, represents a promising therapeutic target. Here, we isolated and characterized three fully human monoclonal antibodies (huMAbs) against SLO (C11, G4, and L17) from the memory B cells of a naturally immunized donor using antigen-specific B-cell enrichment. All three huMAbs bound SLO with high affinity and potently neutralized SLO-induced hemolysis *in vitro*. Mechanistic studies revealed that C11 and G4 inhibited SLO-membrane binding, whereas L17 protected host cells without blocking membrane binding. Despite similar neutralizing potency *in vitro*, only L17 significantly improved disease outcomes *in vivo*. In a murine model of GAS-NSTI, L17 reduced clinical disease severity and prolonged survival by approximately 130%. Together, these findings demonstrate that antibody-mediated neutralization of SLO can alter disease outcomes during GAS-necrotizing infection and support the potential of L17 as a clinically relevant antitoxin therapeutic candidate for GAS-NSTI.

## INTRODUCTION

Necrotizing soft tissue infections (NSTIs) due to *Streptococcus pyogenes* (group A streptococcus [GAS]) remain a devastating cause of morbidity and mortality worldwide (1). Despite modern antibiotic regimens, NSTIs progress rapidly and require urgent surgical intervention, including amputation, to ensure survival (1). Because of the fulminant nature of the disease, broad-spectrum antibiotic therapy must be initiated immediately (2). However, profound tissue ischemia and necrosis characteristic of NSTIs impair regional perfusion, limiting antibiotic delivery and increasing the risk of subinhibitory drug exposure (3, 4). Subinhibitory concentrations not only fail to eradicate the pathogen but can paradoxically exacerbate disease by inducing virulence factor expression (5). Indeed, we and others have demonstrated that subinhibitory antibiotics trigger increased toxin production in defense of antibiotic threat (6, 7). To suppress toxin production, current clinical guidelines for NSTIs recommend a protein-synthesis inhibitor antibiotic (e.g., clindamycin) in combination with cell wall-active antibiotics (e.g., penicillin or vancomycin) (2, 8). However, increasing the rates of clindamycin resistance among GAS isolates (9–11) creates a growing therapeutic dilemma and underscores the need for adjunctive strategies that directly target toxin-mediated pathology.

The mechanisms underlying rapid tissue destruction and systemic collapse during GAS-NSTI are not fully understood, although substantial evidence suggests that the cholesterol-dependent cytolysin (CDC), streptolysin O (SLO), plays a central role (12, 13). At high concentrations, SLO is a potent pore-forming cytolysin that induces extensive cellular damage. Pore formation is a multistep process in which SLO first binds the host cell membrane, oligomerizes on the membrane surface, undergoes a large conformational change to form a pre-pore β-barrel, and then inserts into the lipid bilayer to create large transmembrane pores (14). In lower concentrations, SLO induces platelet-neutrophil co-aggregation, leading to vascular occlusion and subsequent ischemic necrosis (15), and directly impairs cardiomyocyte function (16), potentially contributing to the hemodynamic instability observed in patients with GAS-NSTI (17, 18). Notably, SLO is highly conserved among GAS strains and strongly immunogenic in humans (19). In fact, individuals lacking pre-existing anti-SLO antibody titers appear to be at an increased risk of severe invasive disease (20).

Thus, neutralization of secreted toxins, like SLO, is a potential adjunctive therapeutic strategy. Currently, the only toxin-neutralizing therapy for NSTIs is intravenous immunoglobulin (IVIG) (1, 2). Derived from pooled donor plasma, IVIG has demonstrated efficacy in both human studies and in murine models of NSTI (21, 22). However, its therapeutic efficacy has been inconsistent, largely due to lot-to-lot variability in antitoxin titers and undefined functional potency (23).

Due to their target specificity, defined functionality, and scalability, human monoclonal antibodies (huMAbs) offer several advantages over polyclonal preparations. Despite the rapid expansion of monoclonal antibody therapeutics for cancer and autoimmune diseases, relatively few have been developed for bacterial infections (24). Here, we report the generation and functional characterization of three fully human anti-SLO monoclonal antibodies and evaluate their *in vivo* efficacy. Although all three huMAbs exhibited high affinity and potent *in vitro* neutralizing activity, only one conferred significant protection *in vivo*, prolonging survival and reducing disease severity in a murine model of GAS-NSTI.

## RESULTS

### Generation of anti-SLO huMAbs

**Fig 1:**
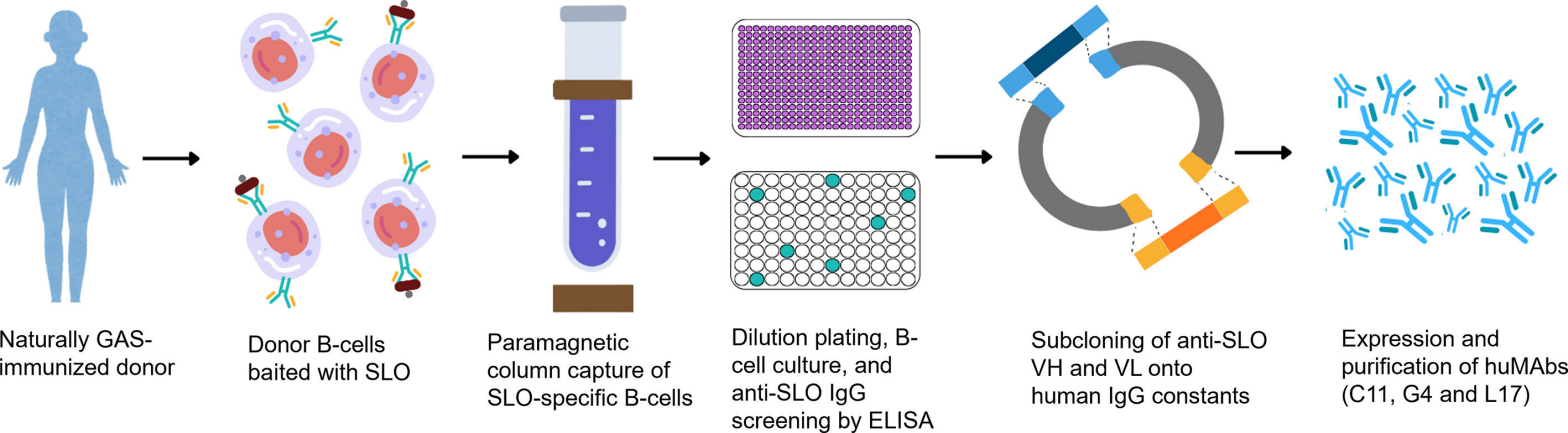
Workflow for the discovery of anti-SLO huMAbs. Peripheral blood was obtained from a donor previously infected with GAS. Antigen-specific B cells were enriched using biotinylated SLO and streptavidin paramagnetic selection. B cells were cultured at limiting dilution and screened for anti-SLO IgG secretion. Variable heavy and light chain genes from positive wells were cloned into expression vectors containing human IgG1 constant heavy and light chains, and recombinant antibodies were expressed and purified for downstream characterization.

Using our antigen-specific B-cell enrichment method (25), followed by B cell culturing, SLO-specificity screening, antibody cloning, and recombinant expression (Fig. 1), we isolated SLO-specific huMAbs from the memory B cells of a donor naturally immunized more than 20 years prior. Three stable huMAbs with high recombinant expression levels were identified: 55C11, 55G4, and 55L17 (herein C11, G4, and L17).

### Anti-SLO huMAbs are SLO-specific and bind SLO with high affinity

A key consideration for therapeutic monoclonal antibodies is target specificity, as off-target binding can result in unintended biological effects (26). Since SLO shares structural and functional similarities with other CDCs (27) and because cross-reactive monoclonal anti-CDC antibodies have been described (28), we assessed binding to other CDCs, perfringolysin O, listeriolysin O, or pneumolysin. Demonstrating high specificity to SLO, none of the huMAbs bound any other CDCs by ELISAs (data not shown).

**Fig 2:**
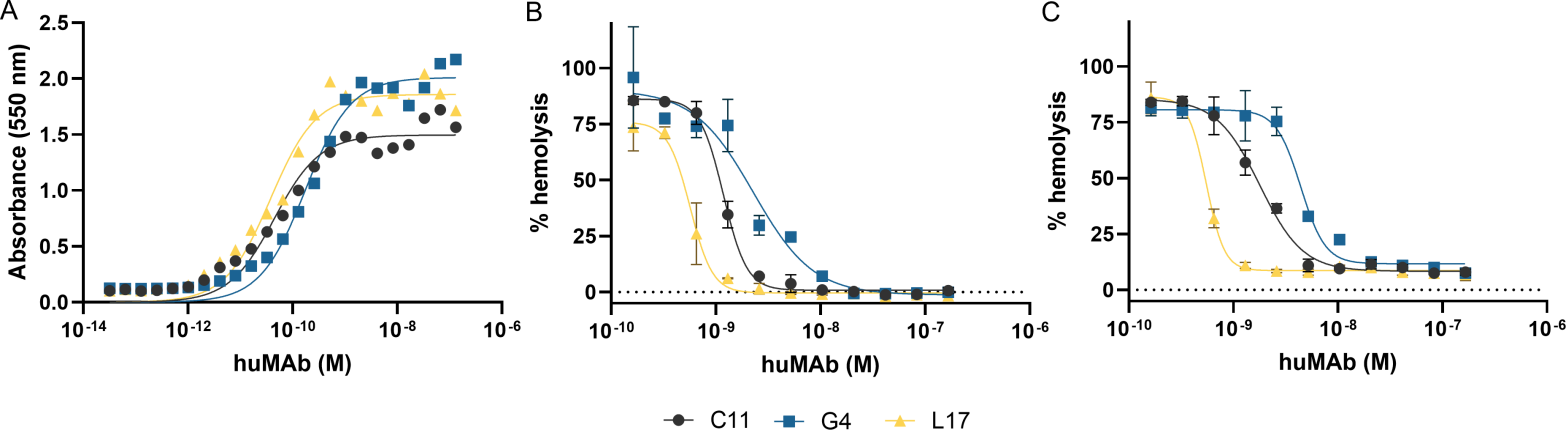
Anti-SLO huMAbs bind and neutralize SLO. (**A**) Binding of C11, G4, and L17 to immobilized SLO was measured by ELISA, and absorbance was plotted as a function of huMAb concentration in molar (M). Apparent binding affinity (KdApp) was determined by nonlinear regression. Curves are representative of *n* = 3 independent experiments. (**B**) Inhibition of recombinant (rSLO)-induced hemolysis. RBCs were incubated with four hemolytic units (HU) of rSLO in the presence of increasing concentrations of huMAbs. (**C**) Inhibition of hemolysis induced by GAS culture supernatants. RBCs were incubated with culture supernatant (equivalent to 8 HU of SLO) in the presence of huMAbs. In (**B and C**), hemolysis was quantified relative to complete lysis controls, IC50s were determined by nonlinear regression, and error bars indicate SD of technical replicates. Data are representative of *n* = 3 (**B**), *n* = 2 (**C**) independent experiments.

Next, the binding affinity to SLO was determined by ELISA. All three huMAbs had high apparent affinity to SLO, with dissociation constants in the mid-picomolar range (Fig. 2A; Table 1), with G4 exhibiting slightly reduced affinity relative to C11 and L17.

**TABLE 1:** Characteristics of anti-SLO huMAbs

huMAb	KdApp (pM)*^a^	IC50 rSLO (nM)**^b^	IC50 supernatant (nM)**^b^
C11	20 ± 19	3.1 ± 5.1	1.9 ± 0.2
G4	115 ± 92	3.4 ± 2.0	7.2 ± 5.2
L17	27 ± 31	1.4 ± 2.2	0.47 ± 0.1

^*a*^ Apparent binding affinity (KdApp), geometric mean (GM) ± standard deviation (SD) in picomolar (pM).

^*b*^ Concentration of 50% inhibition, GM ± SD in nanomolar (nM).

### Anti-SLO huMAbs neutralize recombinant and native SLO

Neutralization of recombinant (rSLO) was evaluated using a red blood cell (RBC) hemolysis assay. All three huMAbs (C11, G4, and L17) potently inhibited rSLO-induced hemolysis in a concentration-dependent manner (Fig. 2B). Nonlinear regression analysis yielded IC50 values in the low nanomolar range (Table 1).

To evaluate activity against natively produced SLO, RBCs were incubated with GAS culture supernatants in the presence or absence of antibody. Culture supernatants induced robust RBC lysis in the absence of huMAb, whereas the addition of anti-SLO huMAbs markedly reduced hemolysis (Fig. 2C). Although all three huMAbs neutralized native SLO with nanomolar potency (Table 1), a 3-fold decrease in the IC50 value was observed for L17 relative to rSLO. These results confirm that huMAbs neutralize naturally produced SLO and remain functionally active in the presence of GAS culture supernatants.

### Anti-SLO huMAbs protect epithelial cells through distinct mechanisms of action

**Fig 3:**
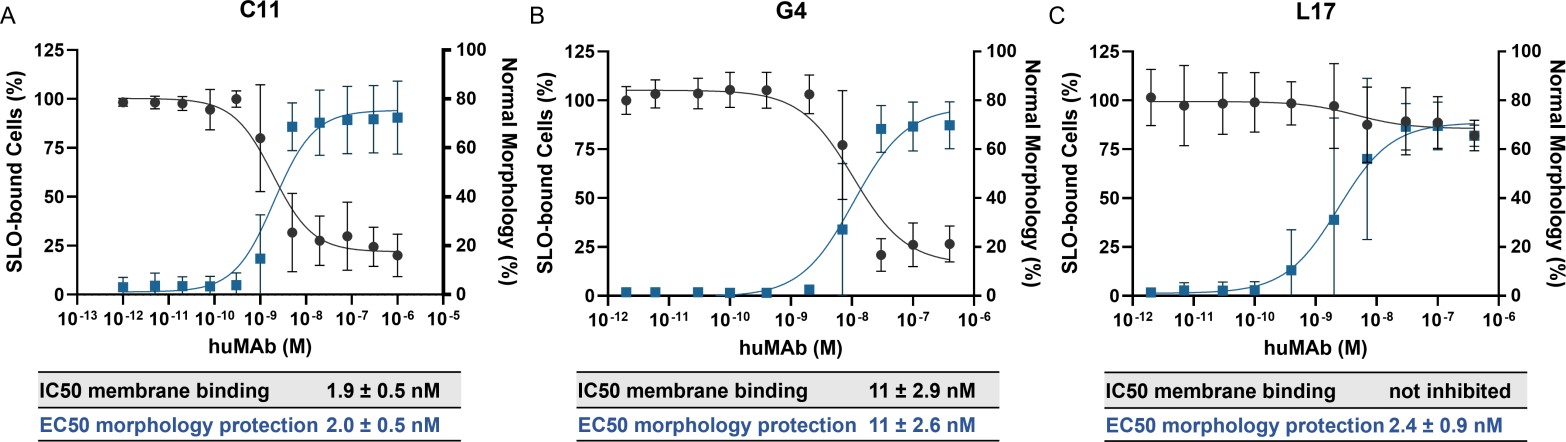
HuMAbs neutralize SLO by distinct mechanisms of action. Fluorescently labeled SLO was incubated with increasing concentrations of C11 (**A**), G4 (**B**), or L17 (**C**), and added to A549 cells. The percentage of SLO + A549 cells (black circles) and the percentage of cells with normal FSC/SSC morphology (blue squares) were determined by flow cytometry and plotted as a function of huMAb concentration. 7-AAD staining confirmed that > 90% of events in the normal-morphology gate were membrane-intact, and SLO + events were predominantly 7-AAD + at the time of acquisition. Symbols represent the mean ± SD from independent experiments (C11, *N* = 6; G4 and L17, *N* = 5). Solid lines indicate nonlinear regression fits used to calculate IC50 values. IC50 values shown below each graph correspond to protection from epithelial cell morphology and inhibition of SLO membrane binding of A549 cells.

To gain insight into the mechanism by which anti-SLO huMAbs protect cells from SLO-mediated damage, we evaluated their effects on SLO membrane binding and preservation of cell integrity (by assessment of morphology) using fluorescently labeled SLO and flow cytometry. C11 and G4 inhibited SLO membrane binding to both RBCs (Fig. S1) and A549 epithelial cells, as shown by a dose-dependent reduction in the percentage of SLO+ cells (Fig. 3A and B, black circles). In parallel, C11 and G4 protected the normal FSC/SSC morphology of epithelial cells with similar IC50 values (Fig. 3A and B, blue squares), consistent with the inhibition of SLO membrane binding as their primary protective mechanism. In contrast, L17 conferred robust protection of epithelial cell morphology (Fig. 3C, blue squares) despite minimal inhibition of SLO membrane binding (Fig. 3C, black circles) across the same concentration range. These findings suggest that L17 neutralizes the pore-forming activity of SLO without preventing membrane binding.

### L17 recognizes a distinct SLO epitope

**Fig 4:**
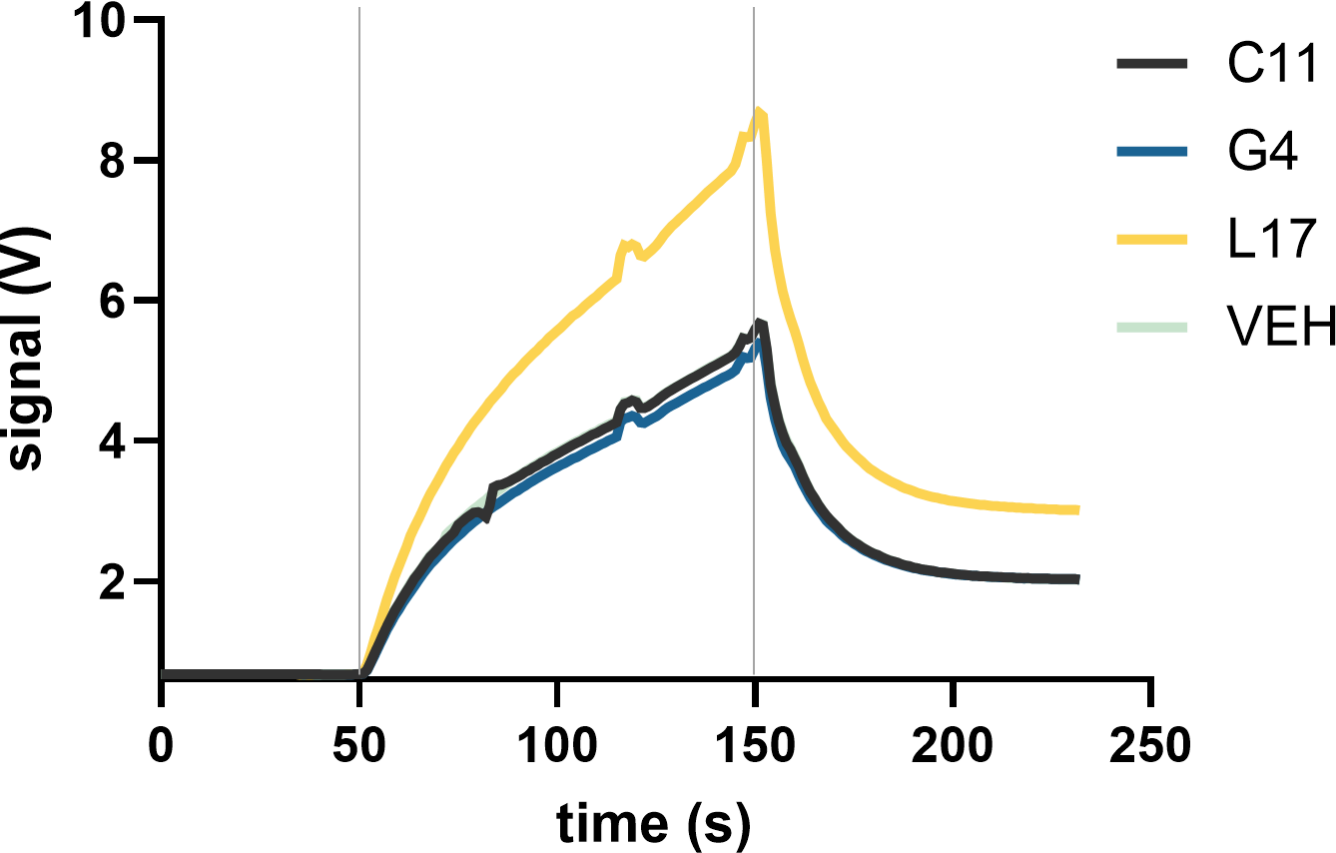
Competitive binding analysis of anti-SLO huMAbs by KinExA. Raw KinExA signal traces showing SLO-C11 complexes with competing (C11 or G4) or non-competing (L17) huMAb or vehicle (VEH). Binding of huMAbs to SLO was detected using an Alexa Fluor 647-conjugated anti-human IgG secondary antibody. Baseline signal was recorded for the first 50 s, followed by the addition of secondary labeling antibody (50–150 s). Excess secondary antibody was removed during the wash phase (150–230 s). The final signal reflects the relative amount of antibody bound to SLO. SLO-coated beads were initially saturated with C11 prior to analysis. Reapplication of C11 serves as a saturation and stability control for the pre-formed SLO-C11 complex. VEH (buffer without huMAb) serves as a non-competition control. Maintenance of signal in the presence of a competing huMAb indicates competitive binding to overlapping epitopes, whereas an increase of signal indicates non-competitive binding consistent with simultaneous binding of multiple huMAbs to distinct epitopes.

Since L17 neutralized SLO without measurably inhibiting membrane binding, we asked whether it recognizes a distinct epitope from C11 and G4. Given that the cholesterol-binding region of SLO is only two amino acids (29, 30), therefore comprising a small area, it is likely that antibodies that inhibit membrane binding have overlapping epitopes. Consistent with this, a competitive binding assay revealed that C11 and G4 were mutually competitive and could not bind SLO simultaneously (Fig. 4). In contrast, L17 and C11 were non-competitive and were able to bind SLO simultaneously, indicating that L17 recognizes a distinct epitope.

### Anti-SLO huMAbs are not synergistic

Previous studies have shown that antibodies recognizing distinct epitopes can exhibit synergistic neutralization activity (31). Therefore, all pairwise combinations of C11, G4, and L17 were evaluated for protective synergy in the RBC hemolysis assay. Combination data were analyzed using the Highest Single Agent (HSA) model in SynergyFinder (32). No huMAb combination improved neutralization beyond the most active single huMAb (Fig. S2), indicating additive rather than synergistic interactions.

### L17 improves survival and reduces disease severity in a murine model of GAS-NSTI

**Fig 5:**
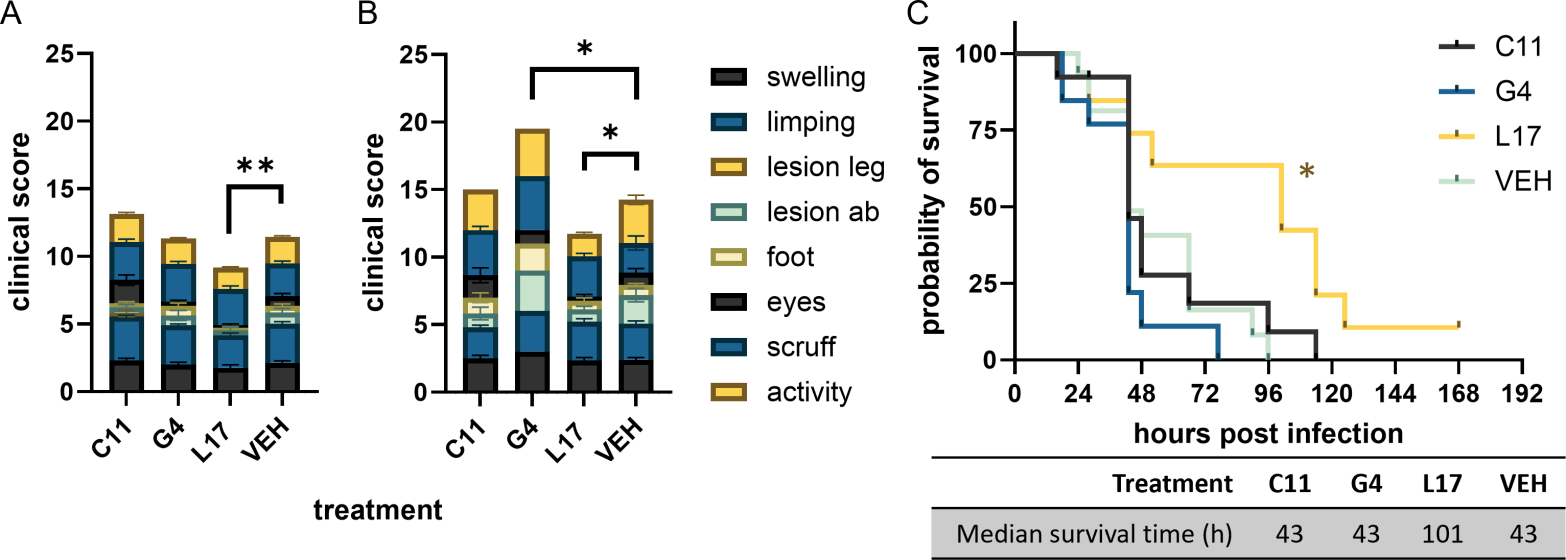
L17 improves symptoms and prolongs survival from GAS-NSTI. Panels A-C represent analyses performed on the same cohort of mice (*n* = 13 per treatment group). Signs of local infection (limb swelling, limping, lesions of the leg or abdomen [abs], and foot blackening) and systemic infection (eye closing and discharge, scruffy fur, and activity) were scored 24 h (**A**) and 48 h (**B**) post-infection. Data are shown as mean ± SEM. Statistical differences were determined by two-way ANOVA followed by Dunnett’s multiple comparisons test; *P ≤ 0.05, **P ≤ 0.01. (**C**) Kaplan-Meier survival curves of adult Swiss Webster mice treated with a single dose of huMAb (25 mg/kg) or vehicle (VEH) 24 h before intramuscular challenge with 3.0 × 10^8^ CFU GAS. *P = 0.014 by log-rank test. The table summarizes median survival times for each treatment group.

Finally, we evaluated whether anti-SLO huMAbs could improve survival and reduce disease severity compared to vehicle-treated controls in a murine model of GAS-induced myonecrosis. Because tissue ischemia is a major barrier to therapeutic delivery in NSTIs, huMAbs were administered 24 h before infection to maximize bioavailability at the time of bacterial challenge. Following infection, all treatment groups rapidly developed clinical signs of GAS disease. However, mice treated with L17 exhibited significantly reduced clinical symptom scores compared to vehicle-treated controls at both 24 and 48 h post-infection, whereas C11- and G4-treated mice showed no improvement (Fig. 5A and B). In support of the symptomatic data, Kaplan-Meier survival analysis demonstrated that L17 significantly prolonged survival compared to vehicle controls, increasing median survival time by approximately 130% (> 2 days; *P* = 0.014 by log-rank test; Fig. 5C). In contrast, C11 and G4 did not confer a survival benefit.

To assess tissue pathology and systemic immune responses during early disease, three mice per treatment group were prospectively selected and euthanized 28 h post-infection. All animals were included in the survival analysis from the time of infection, and animals designated for tissue collection were censored at 28 h. Histological examination of infected muscle revealed extensive necrosis, edema, and inflammatory infiltrates relative to healthy controls, with no discernible differences among treatment groups (Fig. S3). Similarly, spleens from infected mice showed characteristic signs of systemic infection, including increased inflammatory cell infiltration, megakaryocytes, and germinal center formation, without treatment-dependent differences. Minimal or no pathology was observed in the liver, kidney, or heart at this time point.

**Fig 6:**
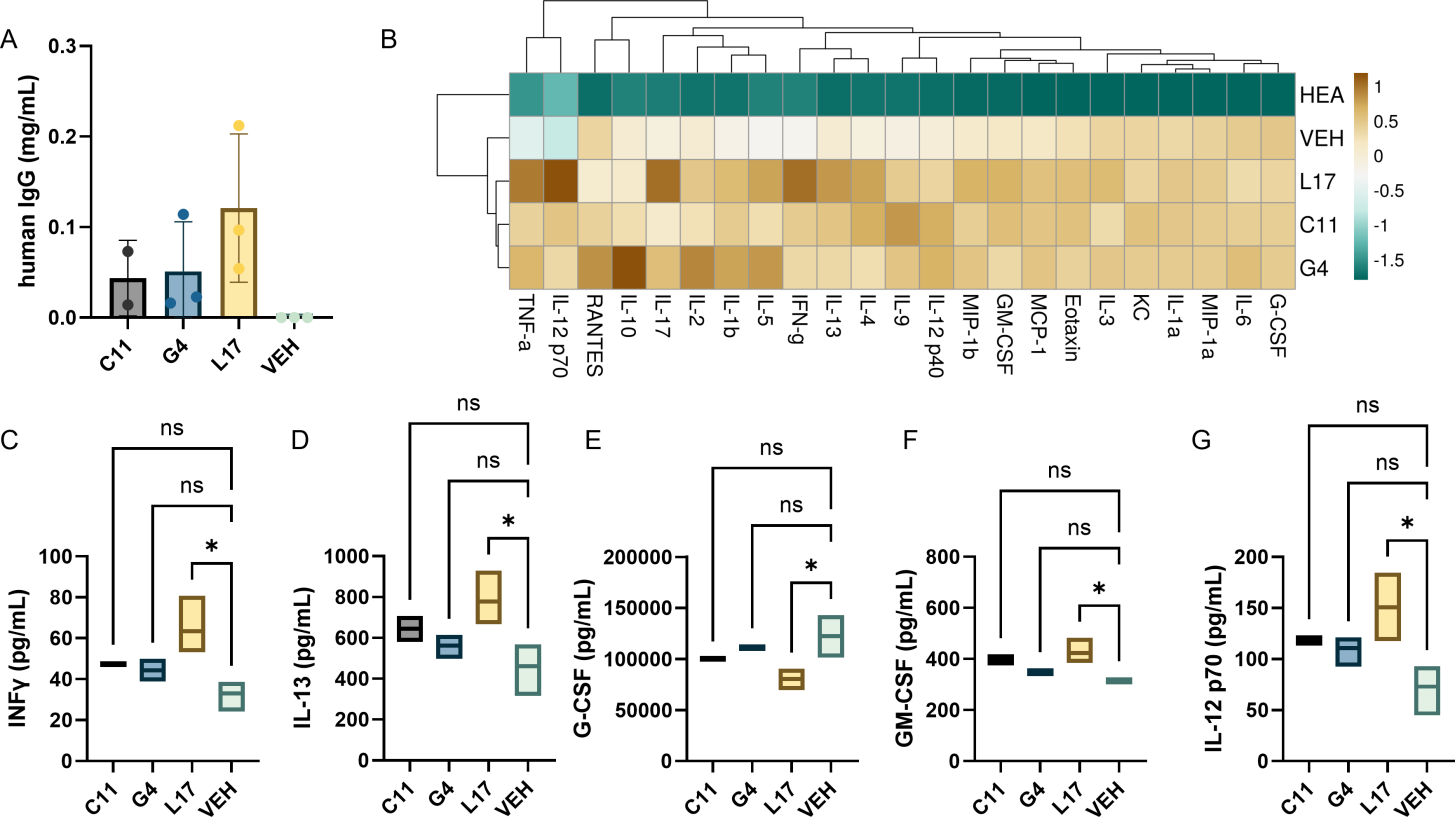
L17 alters serum cytokine responses following GAS infection. (**A**) Serum concentration of huMAbs compared to vehicle (VEH) measured 28 h post-infection. Data shown are mean ± SD (*n* = 2, C11; *n* = 3 G4, L17, and VEH). (**B**) Heatmap of serum cytokine and chemokine concentrations across treatment groups. HEA denotes healthy, non-infected mice. Cytokine values were ln(x)-transformed and visualized by hierarchical clustering. (**C–G**) Box plots showing cytokines with statistically significant differences between treatment groups. Box size indicates max and min. Center lines indicate the mean. Statistical comparisons were performed by one-way ANOVA followed by Dunnett’s multiple-comparison test versus VEH control.

Serum huMAb concentrations were comparable across all treatment groups (Fig. 6A) and were within the expected range based on prior pharmacokinetic studies of human IgG in mice (33, 34). Total endogenous mouse IgG levels varied only slightly between infected and non-infected animals (data not shown). Of the 23 cytokines and chemokines measured, most showed higher concentrations in GAS-infected mice compared to healthy controls, although only a subset reached statistical significance after correcting for multiple comparisons (Fig. S4). HuMAb treatment was associated with broadly increased cytokine levels compared to vehicle controls, with no distinct clustering of treatment groups or cytokine subsets (Fig. 6B). Interestingly, only L17 produced statistically significant differences in individual cytokines, including IFN-γ, IL-13, G-CSF, GM-CSF, and IL-12 p70 (Fig. 6C through G). These data suggest that L17 uniquely alters the host inflammatory response during early GAS-NSTI, although the relationship between cytokine modulation and improved survival remains to be determined.

## DISCUSSION

GAS-NSTIs are among the most devastating forms of invasive bacterial disease and remain associated with substantial morbidity and mortality (1). Therapeutic options beyond surgical intervention are extremely limited (2), and despite being a toxin-mediated disease, no targeted antitoxin therapies currently exist. SLO represents a highly conserved, key virulence factor in GAS-NSTI, directly injuring host cells and disrupting vascular, cardiac, and immune function (13, 18). In this study, we identified three huMAbs that target SLO and potently neutralize toxin-mediated hemolysis *in vitro*. Competitive binding and membrane-binding assays indicate that C11 and G4 have overlapping epitopes and inhibit membrane binding, whereas L17 binds a separate epitope and inhibits SLO activity after membrane association. Despite comparable neutralizing activity *in vitro*, only L17 significantly improved outcomes *in vivo*. Together, these findings support SLO as a promising therapeutic target, identify L17 as a potential clinically relevant antitoxin therapeutic candidate, and demonstrate that *in vitro* toxin neutralization activity alone is insufficient to predict *in vivo* efficacy.

Although toxin neutralization represents an attractive strategy for treating severe GAS infections, current approaches remain limited. IVIG has been used as adjunctive therapy based on its ability to neutralize streptococcal exotoxins (35), but antitoxin titers are variable (23), and clinical outcomes have been inconsistent (22, 36), highlighting the need for more targeted strategies. Alternative approaches, including nanoparticle-based sequestration of SLO, have also been shown to reduce toxin-mediated injury in experimental models (37). As a more defined approach, Matsumura et al. demonstrated that a murine monoclonal antibody (HS1), originally generated against the related CDC perfringolysin O^28^, improved survival in a murine model of streptococcal toxic shock syndrome, providing early proof-of-concept that antibodies targeting CDC toxins can mitigate severe GAS disease (38). In contrast, the huMAbs described here were generated directly against SLO, do not cross-react with related CDCs, and are fully human, supporting their potential for therapeutic development. Importantly, their fully human origin further enhances translational potential by reducing concerns related to immunogenicity. In addition to development as standalone therapeutics, defined anti-SLO huMAbs could potentially be used to supplement IVIG preparations, thereby improving the consistency and potency of SLO-neutralizing activity. Together, these findings extend prior work and further support targeted toxin neutralization as a strategy for invasive GAS infection.

An unexpected finding of this study was that only L17 improved outcomes *in vivo* despite similar neutralizing activity to C11 and G4 *in vitro*. One possible explanation is that antibodies interfering with different stages of pore formation are not functionally equivalent during infection. However, because all three huMAbs had similar IC50 values in all assays that measure pore-mediated cytotoxicity, it seems unlikely that inhibiting different mechanisms of pore formation alone fully explains the observed differences *in vivo*. Another possibility is that the *in vitro* assays performed capture the cytolytic activity of SLO but do not reflect other SLO-mediated effects that contribute to GAS pathogenesis, that is, platelet-neutrophil co-aggregation (15), synergistic interactions with NADase (39, 40), and suppression of neutrophil functions (41, 42). If L17 more effectively blocks these additional SLO-mediated activities, improved protection would be expected. However, this explanation alone is also unlikely to fully account for the observed differences, as prior studies demonstrate that pore-defective SLO mutants retaining certain biologically active activities are insufficient to support full virulence (42–44). Thus, we hypothesize that the superior efficacy of L17 may reflect differences in epitope location or huMAb biophysical properties. For example, antibodies that rely on steric hindrance to block toxin activity may be more susceptible to proteolytic cleavage or structural modification in the complex *in vivo* environment, which could reduce their functional capacity during infection. Alternatively, differences in antibody stability, tissue penetration, or *in vivo* half-life could influence the extent to which SLO is neutralized at sites of infection. Further studies will be required to determine which of these factors contributes to the superior therapeutic efficacy of L17.

Although L17 significantly prolonged survival in this model, protection was not complete, suggesting that neutralization of SLO alone and/or the single-dose regimen may be insufficient to fully terminate GAS virulence during NSTI. GAS produces multiple virulence factors (12) that contribute to disease pathogenesis, and targeting SLO alone may therefore provide only partial protection. In addition, the use of a single-dose strategy in the immunocompetent mice, where human antibodies can exhibit relatively rapid clearance (33), may have limited the duration of effective toxin neutralization. Together, these considerations support the potential for improved outcomes through optimized dosing strategies and/or combination approaches, such as co-administration with antibiotics that suppress toxin production or antibodies targeting additional GAS virulence factors.

Neutralization of SLO with L17 was also associated with changes in the systemic cytokine response during infection. At 28 h post-infection, L17-treated animals exhibited increased levels of IFN-γ, IL-13, GM-CSF, and IL-12p70, while G-CSF levels were reduced. Collectively, this cytokine profile is consistent with activation of immune pathways associated with protection in prior studies of invasive GAS infection (45–47) or other infection types (48). Specifically, IL-12 has been shown to increase resistance to GAS infection in murine models (45), and IFN-γ-producing myeloid cells have been shown to confer protection during severe invasive GAS infection (46). Additionally, the increase in GM-CSF may further support protection by promoting activation and differentiation of myeloid cells, including macrophages, which could enhance bacterial clearance (46). In contrast, the reduction in G-CSF may reflect reduced neutrophil mobilization during infection (47), a process that has been implicated in both host defense and tissue injury in other severe infection models (49). The increase in IL-13, a cytokine associated with anti-inflammatory and tissue-repair processes (48), may indicate that L17 treatment shifts the host response toward recovery. Together, these findings are consistent with the possibility that SLO neutralization influences host immune responses in addition to its direct cytolytic effects. However, the present data do not distinguish whether these cytokine changes arise directly from SLO neutralization or occur secondarily as a consequence of reduced tissue injury or improved infection control.

A limitation of this study is that the huMAbs were administered prophylactically prior to infection. While this does not fully reflect the clinical scenario in which treatment is initiated after disease onset, this approach enables evaluation of the intrinsic protective capacity of each antibody *in vivo* while minimizing variability introduced by differences in the timing of disease progression. Thus, these findings provide a clear rationale for future studies evaluating post-infection dosing strategies and direct comparison with IVIG in clinically relevant models.

In summary, this study provides proof-of-concept that antibody-mediated neutralization of SLO can improve disease outcomes in a preclinical model of GAS-NSTI and identifies a fully human monoclonal antibody with therapeutic potential. Importantly, we also show that antibodies with comparable *in vitro* neutralizing activity can differ markedly in their *in vivo* efficacy, indicating that *in vitro* assays alone may not fully capture the properties required for efficacy during infection. The partial protection conferred by L17 likely reflects a combination of factors, including dose regimen and the complex, multifactorial nature of GAS pathogenesis. Ultimately, these results support further development of optimized antibody-based strategies to improve outcomes in severe invasive infections.

## MATERIALS AND METHODS

### Antigen expression and modifications

Oxygen-stable SLO (SLOwt; GenBank accession P0DF96 [50]), cloned into the pTrcHiS vector, was kindly provided by Rodney K. Tweten (University of Oklahoma Health Sciences Center, Oklahoma City, OK). To perform high-specificity isolation while preserving native antigenic epitopes, SLOwt was modified at the N-terminus to include a His-tag (His6), an AviTag for mono-biotinylation, and a flexible glycine-serine linker, as we have previously described (25). To prevent cholesterol-dependent membrane binding during B cell enrichment, point mutations were introduced into the cholesterol recognition motif (T564G and L565G; herein SLOm) using Q5 site-directed mutagenesis (New England BioLabs) and confirmed by DNA sequencing (MCLAB). Recombinant His-tagged SLO and derivatives were expressed in *E. coli* Rosetta DE3 pLysS cells and purified as we described in a previous study (25). Purified SLOwt and SLOm were stored at −80°C in freezing buffer (50 mM HEPES, pH 7.5, 100 mM NaCl, 1 mM EDTA, 1 mM TCEP, 0.02% NaN3, 10% glycerol). Endogenous mono-biotinylation was verified by anti-biotin ELISA.

### Antigen-specific B cell enrichment

This study was approved by the Veterans Administration Puget Sound Internal Review Board (IRB #00923), and written informed consent was obtained from donors with prior mild-to-severe GAS infection. Peripheral blood was collected in heparinized tubes, and anti-SLO (ASO) titers were determined by the Boise VA Medical Center clinical laboratory to confirm natural immunization status. Peripheral blood mononuclear cells were isolated by Ficoll-Paque density centrifugation, as we have previously described (25).

B cells were enriched by depletion of non-B cells and IgM + B cells using magnetic bead-based negative selection (Miltenyi Biotec). Antigen-specific enrichment was performed by incubating pre-enriched B cells with 6.25 nM mono-biotinylated SLOm, followed by positive selection using streptavidin-coated magnetic beads and MACS columns (Miltenyi Biotec), as we have previously described (25). The resulting SLO-specific B cell population was eluted for downstream culture.

### Propagation and identification of antigen-specific B cells

Enriched SLO-specific B cells were cultured in 384-well plates with irradiated 3T3-msCD40L feeder cells, IMDM supplemented with 10% heat inactivated FBS, IL-2, IL-21, and MycoZap, as we have previously described (25). Cells were plated at limiting dilution and cultured for 12 days. Supernatants were harvested for screening of anti-SLO production by ELISA, and B cells from anti-SLO-positive wells were lysed for cloning.

### Anti-SLO IgG ELISA

To quantify IgG, high-binding 96-well plates were coated with 0.5 µg/mL unconjugated goat anti-human IgG Fc capture antibody (Invitrogen, Thermo Fisher Scientific). To quantify antibodies binding SLO, streptavidin-coated 96-well plates were incubated with biotinylated SLOwt at 37°C for 1 h. Cell culture supernatants were diluted in sample buffer (PBS plus 5% non-fat dry milk) and incubated on plates for 1 h at 37°C. Horseradish peroxidase conjugated goat anti-human IgG antibody (Thermo Scientific) was used for detection, and 1-Step Ultra TMB-ELISA Substrate solution (Thermo Scientific) was used to detect horseradish peroxidase activity according to the manufacturer’s protocol. Reaction was stopped with the addition of 2 M sulfuric acid, and absorbance was read at 450 nm.

### Human antibody cloning and production

Antibody variable region cDNA was generated from antigen-positive B cells using a modified single-cell RT-PCR workflow based on published protocols (51–53). Variable heavy- and light-chain genes were amplified by nested PCR, sequence-verified, and cloned into human IgG1κ or IgG1λ expression vectors (pVITRO) (54) using Gibson assembly. Primer sequences were derived from published sources (51, 53, 54), with minor modifications, and are available from the corresponding author upon request.

Recombinant huMAbs were expressed in Expi293F HEK cells (Thermo Fisher Scientific) following manufacturer protocols, and culture supernatants were harvested seven days post-transfection.

### Determination of binding affinity

Antibody binding affinity to SLO was determined by ELISA. Briefly, streptavidin-coated 96-well plates were incubated with biotinylated SLO at 37°C for 1 h. Plates were washed and incubated with serial dilutions of anti-SLO huMAbs prepared in PBS plus 5% non-fat dry milk and incubated for 1 h at 37°C. Bound huMAbs were detected using horseradish peroxidase conjugated goat anti-human IgG antibody (Thermo Scientific), followed by the addition of 1-Step Ultra TMB-ELISA substrate (Thermo Scientific), according to the manufacturer’s instructions. Reactions were stopped with 2 M sulfuric acid, and absorbance was measured at 450 nm. Equilibrium dissociation constants (Kds) were determined by nonlinear regression fitting using the equation: Y = Bmax*X/(Kd + X), where X represents antibody concentration, Y represents absorbance, and Bmax represents maximum binding.

### Hemolysis neutralization assay

Neutralization of SLO-mediated RBC lysis was evaluated using a standard hemolysis assay. Hemolytic units (HU) were defined as the amount of SLO required to produce 50% lysis of a 2% suspension of sheep RBCs under the assay conditions. Briefly, in a 96-well plate, 4–8 HU of recombinant SLO diluted in PBS or an equivalent volume of GAS culture supernatant from 16 h cultures, was incubated with PBS or increasing concentration of huMAbs (2-fold serial dilutions from 800 to 0.1 nM in PBS) in a final volume of 25 μL for 15 min at 37°C. Following incubation, 50 µL of 2% suspensions of washed sheep RBCs (Hardy Diagnostics) were added to each well, and plates were incubated for an additional 30 min at 37°C. Non-lysed RBCs were pelleted by centrifugation at 1,000 × *g* for 10 min, and supernatants were transferred to a fresh 96-well plate. Hemolysis was quantified by measuring absorbance at 550 nm. Percent hemolysis was determined by normalization to 100% and 0% hemolysis controls, generated by treated RBCs with water or PBS, respectively. The IC50 was determined by nonlinear regression: Y = Bottom + (Top-Bottom)/(1+(IC50/X)^HillSlope), where Top and Bottom represent the response plateaus.

### Prevention of SLO membrane binding and epithelial cell protection

Recombinant SLO, containing the N-terminal mono-biotinylation, was fluorescently labeled via tetramerization with phycoerythrin (PE)-conjugated streptavidin, as we have previously described (25). Briefly, mono-biotinylated SLO was incubated with PE-streptavidin at a molar ratio of 4:1 (SLO:streptavidin) for 60 min on ice to generate fluorescent SLO tetramers. Excess unbound SLO was removed using 100 kDa molecular-weight-cutoff centrifugal filters. Fluorescent SLO tetramers were prepared fresh for each experiment.

For the assessment of membrane binding and epithelial cell protection, 50 nM of fluorescently labeled SLO tetramers was incubated with increasing concentrations of huMAbs (2-fold serial dilutions) in a final volume of 100 μL for 60 min on ice. Following antibody-SLO incubation, 1 × 10^6^ PBS-washed red blood cells or A549 epithelial cells were added and incubated for an additional 60 min on ice. For experiments assessing membrane permeability, 7-aminoactinomycin D (7-AAD; ~1 µL of 1 mg/mL stock) was added during the final 15 min of incubation. Cells were analyzed immediately by flow cytometry (CytoFLEX, Beckman Coulter) without washing. SLO binding was quantified as the percentage of PE-positive cells within the debris-excluded population. Forward and side scatter parameters were used to assess cell morphology, and preservation of normal FSC/SSC profiles was used as a phenotypic measure of epithelial cell integrity. Dose-response curves for membrane binding or epithelial cell morphology protection assays were calculated by nonlinear regression: Y = Bottom + (Top-Bottom)*X/(EC50 +X). For inhibition assays, the midpoint is reported as IC50; for protection assays, it is reported as EC50.

### Competitive antibody binding analysis by KinExA

Competitive binding between anti-SLO huMAbs was evaluated using a KinExA-based bead assay. Briefly, 200 mg of polymethyl methacrylate (PMMA) beads were suspended in 1 mL PBS containing recombinant SLO and incubated for 2 h at room temperature with rotation to allow immobilization of SLO on the bead surface. Beads were washed five times with PBS to remove unbound SLO and subsequently blocked with 10 mg/mL bovine serum albumin in PBS for 2 h at room temperature to minimize non-specific binding. Following blocking, beads were incubated with an excess concentration of C11 to saturate available SLO-binding sites. Bead-SLO-C11 complexes were washed four times with PBS immediately before KinExA analysis with the KinExA 3200 with autosampler (Sapidyne Instruments). SLO bound by C11 was detected using an Alexa Fluor 647-conjugated anti-human secondary antibody at a fixed concentration. Baseline fluorescence was established by flowing secondary antibody alone over SLO-coated beads. To confirm the stability of immobilized C11-SLO complexes, 500 μL of C11 was flowed over the beads, followed by secondary antibody detection. Competitive binding was assessed by flowing 500 μL of a second anti-SLO huMAb (G4 or L17) over C11-SLO-bound beads, followed by secondary antibody detection. If G4 or L17 competed with C11 for the same or overlapping epitopes, no increase in fluorescent signal was observed, indicating replacement rather than additive antibody binding. In contrast, an increase in fluorescent signal indicated non-competitive binding, consistent with simultaneous binding of multiple antibodies to distinct SLO epitopes.

### Measurement of antibody synergy

Synergistic neutralization between anti-SLO huMAbs was evaluated using the hemolysis assay. Briefly, seven 2-fold serial dilutions of each antibody (starting at 42 nM) were combined in a matrix by mixing each concentration of antibody A with each concentration of antibody B in 96-well plates. Percent neutralization was calculated relative to SLO-only and no-SLO controls. Data were analyzed using SynergyFinder+ (32) applying the HSA model to determine whether the combination activity exceeded that of the most active individual huMAb at corresponding concentrations.

### Survival studies

*S. pyogenes* strain ATCC 12384 was isolated on 5% sheep blood agar plates (Hardy Diagnostic) and incubated at 37°C in 5% CO_2_. A single colony was inoculated in 10 mL Todd-Hewitt broth supplemented with yeast extract (THY) and incubated for 13 h with shaking at 100 rpm; 1 mL of the overnight culture was subcultured into 9 mL fresh THY and incubated for an additional 4.5 h under the same conditions. Cultures were washed twice with sterile PBS by centrifugation at 4,000 × *g* for 10 min at 4°C, and the bacterial pellets were resuspended and diluted in sterile PBS to the desired inoculum.

All animal experiments were approved by the Boise VA Medical Center IACUC and were conducted in an AAALAC-accredited facility. Seven-week-old female Swiss Webster mice (Taconic, Rensselaer, NY) were given a single intraperitoneal dose of huMAb (25 mg/kg huMAb; *n* = 13 per huMAb) or vehicle (*n* = 16) 24 h before challenge with 3.4 × 10^8^ CFU of washed GAS that was injected into the right thigh muscle. Mice were monitored daily for signs of local infection (limb swelling, limping, lesions of the leg or abdomen, and foot blackening) and systemic infection (eye discharge and closure, scruffy fur, and activity). Clinical scores ranged from 0 (no symptoms) to 4 (severe symptoms). Clinical scores were analyzed by two-way ANOVA with treatment and time as factors, followed by Dunnett’s multiple-comparison test to vehicle control. Survival was monitored three times daily for 7 days post-infection, and Kaplan-Meier plots and log-rank tests were used for comparison of survival between groups.

To assess early tissue pathology and systemic immune responses, three mice per treatment group were randomly selected prior to infection and euthanized 28 h after infection for blood and tissue collection. All animals were included in the survival analyses from the time of infection. Mice euthanized for tissue collection were censored; thus, they did not contribute to survival data beyond the time of euthanasia. Three non-infected, healthy mice were included for comparison. Blood (~1 mL) was obtained by cardiac puncture and was allowed to clot prior to centrifugation at 1,000 × *g* for the collection of the serum. Serum samples were stored at −80°C until analysis. Muscle from infected and contralateral thighs, spleen, liver, kidney, and heart were fixed in 10% neutral buffered formalin, embedded in paraffin, sectioned (4 µm), and stained with hematoxylin-eosin. Pathology was assessed by a blinded pathologist and staff scientist for edema, inflammation, tissue necrosis, and hemorrhage, on a semi-quantitative scoring rubric (0–4) to guide descriptive assessment of tissue pathology.

### Analysis of huMAbs and cytokines in animal serum

Serum huMAb and total mouse IgG were quantified by ELISA using standard methods and extrapolated from matched standard curves. Samples were analyzed in 2-fold dilutions beginning at 1:10,000. Wells with absorbance of 0.05 and 0.6 were averaged for each animal. Differences between treatment groups were compared by one-way ANOVA and Dunnett’s multiple-comparison test.

Cytokines and chemokines were quantified using the preconfigured Bio-Plex Pro Mouse Cytokine 23-plex Assay (BioRad) according to the manufacturer’s instructions. Serum (25 μL per well) was analyzed on a MAGPIX Luminex system (Thermo Fisher), and concentrations of cytokines and chemokines were calculated using xPOTENT Software Solutions for Luminex (Thermo Fisher). For visual comparison, cytokine data were ln(x)-transformed and analyzed by hierarchical clustering using ClustVis (55). Unit variance scaling was applied to rows, and clustering was performed using Euclidean distance with average linkage. For the analysis of statistical differences, treatment groups were compared by one-way ANOVA and Dunnett’s multiple-comparison test. Cytokine levels in vehicle-treated infected mice were compared to healthy, non-infected mice by unpaired *t*-tests with False Discovery Rate correction using a two-stage step-up method (Fig. S3).

### Data analysis

All statistical analyses were performed using GraphPad Prism 10 software. Statistical tests are specified in the description of each method. Unless otherwise noted, statistical significance was quantified in all tests as *P <  0.05, **P <  0.01, ***P <  0.001, and ****P <  0.0001.

## SUPPLEMENTAL MATERIAL

Supplemental figures aac.00575-26-s0001.pdf Fig. S1 to S4.

ASM does not own the copyrights to Supplemental Material that may be linked to, or accessed through, an article. The authors have granted ASM a non-exclusive, world-wide license to publish the Supplemental Material files. Please contact the corresponding author directly for reuse.
