# Supplementary material for: A fully human streptolysin O-neutralizing monoclonal antibody improves survival in a murine model of group A streptococcus necrotizing soft tissue infection
Source: Antimicrob Agents Chemother. 2026 Jul 17;70(8):e00575-26. doi: 10.1128/aac.00575-26 (PMC13435956; doi:10.1128/aac.00575-26)
Supplement: Supplementary file 1 [file aac.00575-26-s0001.pdf]

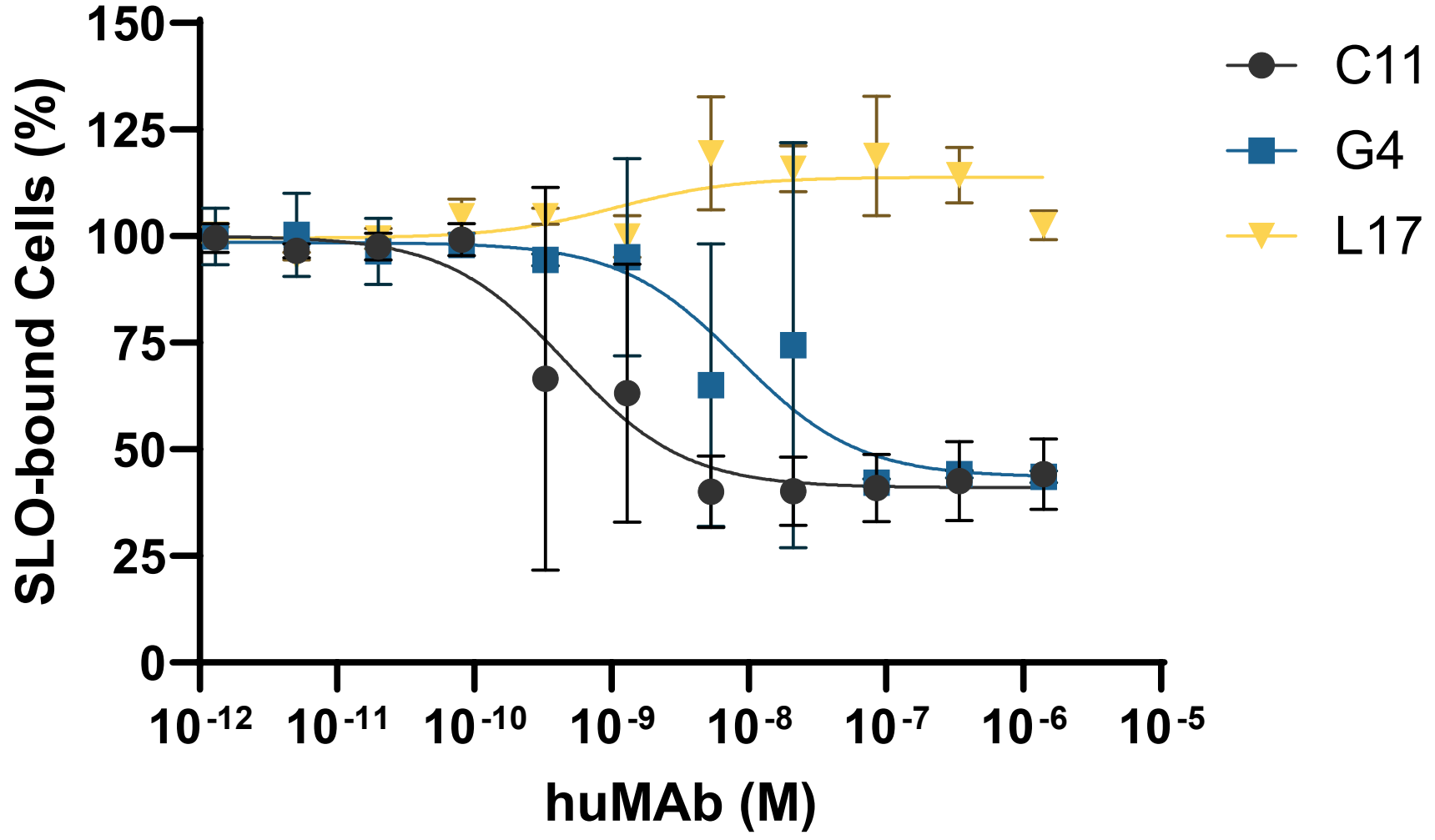

huMAb	IC50
**C11**	**0.5 ± 0.2 nM**
**G4**	**8.3 ± 6.3 nM**
**L17**	**not inhibited**

Supplementary Figure 1. HuMAbs neutralize SLO by distinct mechanisms of action. Fluorescently labeled SLO was incubated with increasing concentrations of C11, G4, or L17 and added to RBCs. The percentage of SLO+ RBCs cells was determined by flow cytometry and plotted as a function of huMAb concentration. Symbols represent the mean ± SD from independent experiments (C11, n=3; G4 and L17, n=2). Solid lines indicate nonlinear regression fits used to calculate IC50 values as reported in the table.

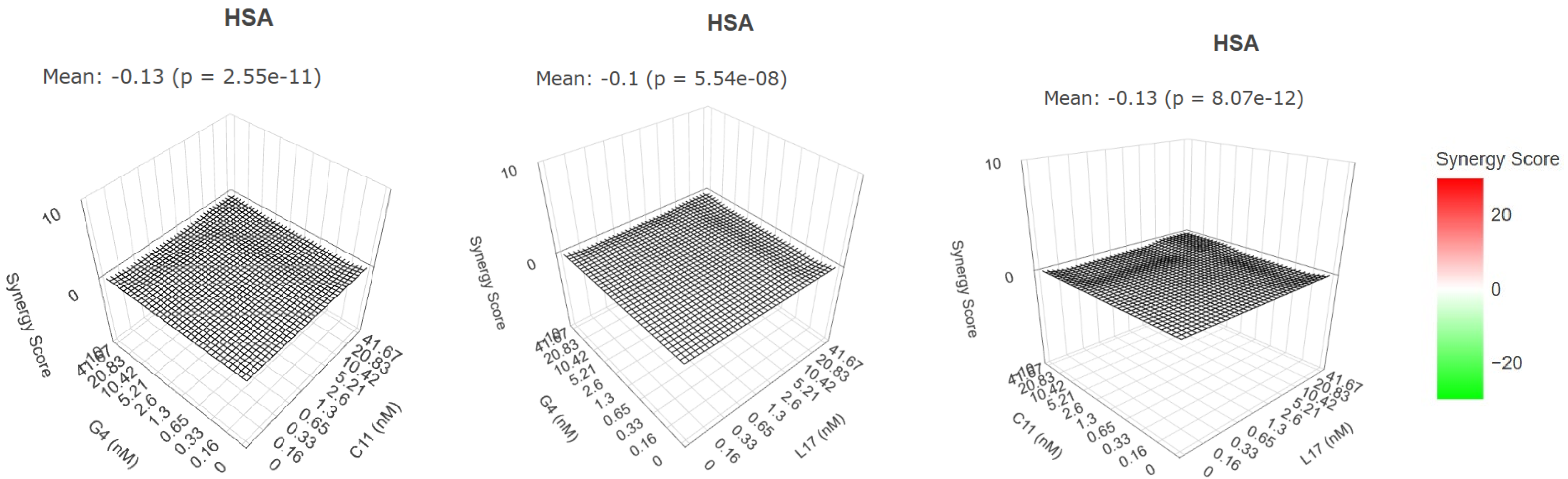

Supplementary Figure 2. Synergy analysis of anti-SLO huMAbs using Highest Single Agent (HAS) model. Synergy scores were calculated from hemolysis inhibition assays performed with pairwise combinations of C11 and G4 (left), G4 and L17 (center), or C11 and L17 (right) across a matrix of antibody concentrations. Three-dimensional surface plots show HSA synergy scores generated using SynergyFinder[32]. Positive scores indicate synergistic interactions, negative scores indicate antagonistic interactions, and scores near zero indicate additive effects. Mean HSA synergy scores above each graph, for all three huMAb combinations, were near zero, indicating that no antibody combination enhanced neutralization beyond the activity of the most effective single huMAb. These results demonstrate additive rather than synergistic interactions among the anti-SLO huMAbs tested.

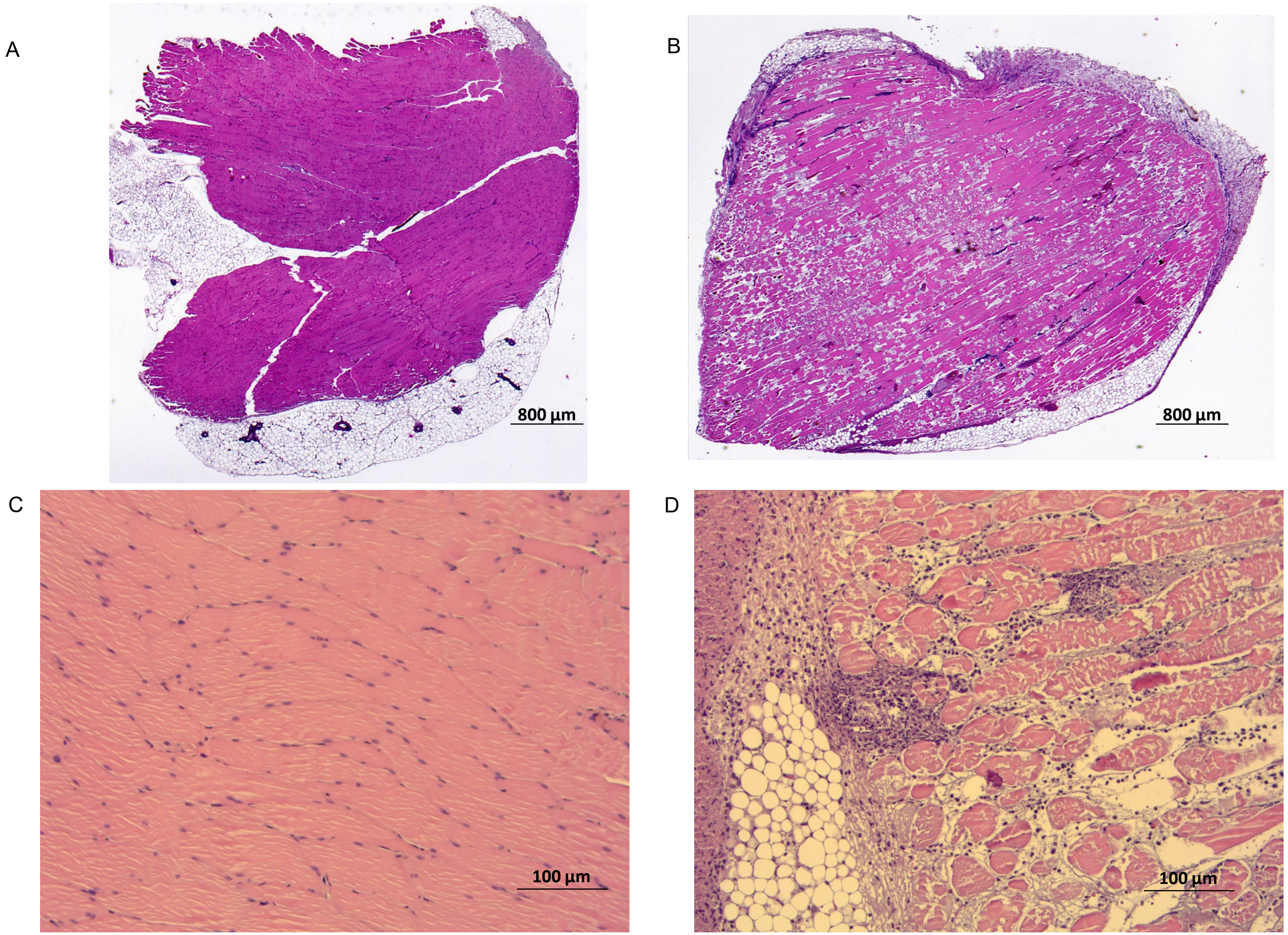

Supplementary Figure 3. Representative histopathology of muscle following GAS infection. H&E-stained sections (4 µm) of the muscle collected 28 h post-infection. Healthy muscle shows intact myofiber architecture (A, C), whereases GAS-infected muscle demonstrates extensive myofiber necrosis, inflammatory infiltrates and edema (B and D). Images shown at 10x (A and B) and 100x (C and D) magnification. Scale bars: 500 um (A and B) and 100 um (C and D).

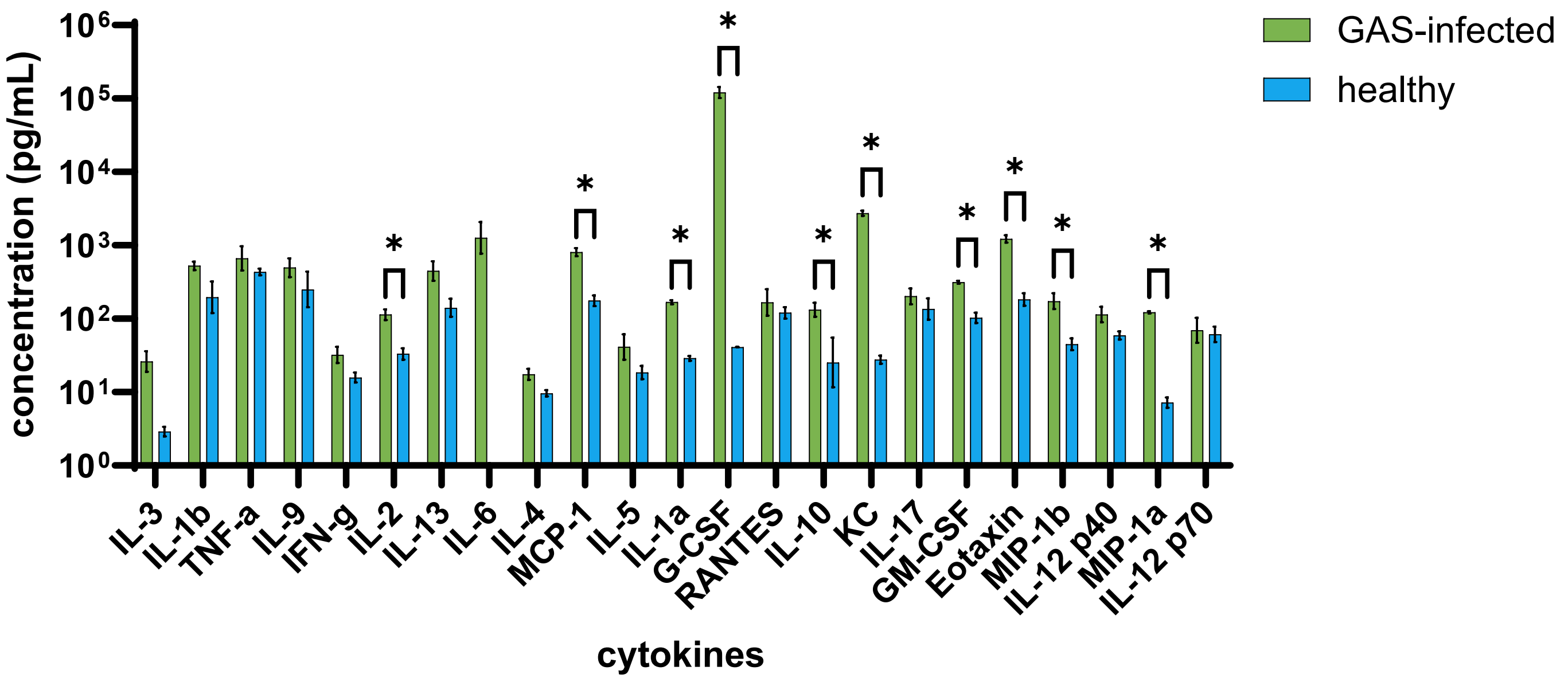

Supplementary Figure 4. Cytokine responses in healthy and GAS-infected mice. Serum cytokine and chemokine concentrations were measured 28 h after infection in healthy mice and vehicle-treated infected mice using a multiplex cytokine assay. Bars represent the geometric mean ± geometric SD. Cytokines were compared by unpaired t-test with false discovery rate correction using a two-stage step-up method.
